# Emotional Safety in the Context of Dementia: A Multiperspective Qualitative Study

**DOI:** 10.3233/JAD-201110

**Published:** 2021-01-05

**Authors:** Silke Kuske, Sandra Olivia Borgmann, Florian Wolf, Christian Bleck

**Affiliations:** a Fliedner Fachhochschule Düsseldorf, University of Applied Sciences, Düsseldorf, Germany; b Hochschule Düsseldorf, Düsseldorf, Germany

**Keywords:** Alzheimer’s disease, dementia, emotion, patient safety, qualitative research, safety

## Abstract

**Background:**

Current research acknowledges the relevance of the emotional safety of people living with dementia. However, available evidence regarding this topic is limited. A comprehensive view of this topic that equally considers the perspectives of people living in an early stage of dementia, relatives, and public stakeholders is lacking.

**Objective:**

This study aimed to obtain a multiperspective view of emotional safety in the context of dementia in the living environment.

**Methods:**

A descriptive qualitative study was conducted based on data collected through semi-structured guided interviews (*n* = 14), focus groups (*n* = 3), guided feedback, and participatory approaches. People living in an early stage of dementia (N = 6), relatives of people living with dementia (N = 11), and public stakeholders (N = 15) were included.

**Results:**

Considering “social togetherness”, “personal condition”, “health”, “physical environment”, and “society” in the light of “living and learning in relations” are preconditions for understanding emotional safety in the context of dementia. “Living and learning in relations” refers to the interaction of people in the context of dementia and relations to the topic of dementia. The focus lies on the (collective) learning. The individuality of each person and his or her situation is central, related to dementia-related, psychosocial, biographical, physical, and economic factors.

**Conclusion:**

Our study highlights the relevance of research on emotional safety in the context of dementia. Approaches to improving the emotional safety of people living in an early stage of dementia should consider the complex situations of each target group in relation to each other at the micro, meso, and macro levels.

## 1 INTRODUCTION

Feeling safe is a primary psychological need of people living with dementia (PlwD) because of their increased general vulnerability and reduced emotio-nal safety [1]. Living with a chronic disease often has a strong impact on coping with changes, and several community or home related entities are involved [2]. Therefore, simultaneously considering emotional, physical, social, and functional safety is essential. It has been indicated that the dimensions of safety are interrelated, and more research regarding the “types and patterns” of safety among all stakeholders is needed [2]. Heyhoe and Lawton (2020) also discuss the impact of emotions in the context of health care delivery, especially on patient safety [3]. However, knowledge regarding the dimensions of emotional safety considering multiple perspectives in the living environment in the context of dementia is limited.

Feeling safe is identified as a main outcome in the community and plays an important role in studies investigating non-pharmacological community-based health and social care [4]. Failure to consider emotional safety could lead to emotional distress, which PlwD can experience as feelings of fear or loneliness [5]. Consistent with the current literature [2, 6– 10], emotional safety in the context of dementia is defined as “the experience of people [in the context of dementia] (...) on a continuum between feeling safe and feeling threatened in the context of subjectively perceived inner and outer conditions” [1]. Emotional safety “refers to the psychological impact of receiving/providing services” [2]. Emotional safety in the context of dementia can be influenced by several factors [1], e.g., psycholo-gical, disease-related, biographical, demographic, socioeconomic, and environmental (social and phy-sical). Therefore, a more comprehensive view of emotional safety is needed as a part of a multiperspective approach involving PlwD, relatives of PlwD, and public stakeholders.

A recent study by Häikiö et al. (2019) showed that preventing physical, economic, relational, and emotional harm to PlwD is among the most important tasks in a community setting [11]. The authors identify a broad range of safety risks and describe themes related to preventing emotional harm, including ”respect and dignity”, “preventing loneliness”, “avoiding negative feelings”, and “promoting good moments and positive feelings” [11]. Although the current research acknowledges the relevance of emotional safety, particularly in relation to dementia, our recent review revealed that limited evidence is available regarding emotional safety in the context of dementia [1]. Additionally, a comprehensive view that equally considers the perspectives of PlwD, relatives of PlwD, and public stakeholders that compares the different experiences of relevant groups in local contexts is lacking. Therefore, the aim of this study was to obtain a multiperspective and comprehensive view of emotional safety in the context of dementia in the living environment.

## 2 MATERIALS AND METHODS

This qualitative research study is a part of the model project “Emotional safety in the context of dementia” conducted between July 2017 and June 2020 as a collaboration between a research team at a University of Applied Sciences and a team from a non-statutory welfare association consulting in the community. This study followed scientific quality standards in qualitative research [12, 13].

### 2.1 Study design and setting

A descriptive qualitative study was conducted based on semi-structured guided interviews, focus groups, guided feedback, and data collected through participatory approaches. A predominately inductive content analysis design was applied [12]. The qualitative study was performed in North-Rhine-Westphalia, Germany and included participants from different communities. People in an early stage of dementia, relatives of PlwD, and public stakeholders were included to ensure a multiperspective and comprehensive view of emotional safety in the context of dementia.

### 2.2 Sampling design, recruitment, and retention

A qualitative research design with a criterion-based convenience and snowball sampling was chosen [14]. The inclusion criteria for all target groups were as follows: 18 years of age or older, living in North Rhine-Westphalia, Germany, and authority to provide informed consent for study participation. Further-more, people at an early stage of dementia (self-re-ported or confirmed diagnosis), relatives of PlwD, and public stakeholders who encountered dementia in their personal and/or professional backgrounds were included.

The participants were recruited between November 2017 and March 2019 predominately by the team of the non-statutory welfare association and partially by the research team. The number of interviews was calculated by considering a community approach, and data saturation was expected to be re-ached [15]. To obtain a more typical sample, we recruited PlwD from community services rather than dementia research centers, although advantages were expected based on Cridland et al. (2016) [16].

The participants were recruited through personal contact, flyers, newspaper articles, and participatory strategies (e.g., communication boards in market-places and thematic exchanges at breakfast meetings). The participants, especially the PlwD, were approached face-to-face or by telephone or mail to establish a relationship of trust between the parties participating in the project to ensure an understanding of the phenomenon of interest and knowledgeability and enhance participant retention [16, 17]. The team members of the non-statutory welfare association invited interested parties to engage in voluntary conversations regarding the project objectives and topics in person or over the telephone.

The following three different approaches were used to confirm the diagnosis of early dementia of PlwD: self-reported dementia, self-reported diagno-sis of dementia, and/or a transmitted document of diagnosis. A self-reported diagnosis was verified by the staff of the local non-statutory welfare associ-ation. Each PlwD was offered an voluntary Mini-Mental State Examination (MMSE). Performing an MMSE was not an inclusion criterion.

### 2.3 Participants

Our study is based on a balanced and comprehensive sample design involving a total of 32 interviewed participants (Table 1). Fourteen semi-structured individual interviews with PlwD (N = 5), relatives of PlwD (N = 7), and public stakeholders (N = 4), including two dyadic interviews (PlwD were attended by their relatives), were conducted. None of the participants of the individual interviews participated twice in interviews. All PlwD had the opportunity to participate in all types of interviews. One interviewed PlwD also participated in a focus group. Three focus groups involving 17 participants, including PlwD (N = 2), relatives (N = 4), and public stakeholders (N = 11), were conducted. One participant represented the perspective of a public stakeholder and the perspective of a person with a suspected diagnosis of dementia. Three participants participated as public stakeholders but were also relatives of people with neurodegenerative diseases (two with dementia and one with another disease). Data saturation was reached; thus, the researchers assumed that further data collection would lead to similar results [15].

**Table 1:** Participants’ characteristics

		Total *n* (% )		Individual interviews		Dyadic interviews	Focus groups
		/ M±SD		n (% ) / M±SD		n (% ) / M±SD	n (% ) / M±SD
			PlwD	Relatives	Public stakeholders
Total number of participants ^(a) ^		32 (100.0)	3 (100.0) ^(a) ^	5 (100.0)	4 (100.0)	4 (100.0)	17 (100.0) ^(a) ^
Participants (N = 32)	People living with dementia ^(a) ^	6 (18.8)	3 (100.0) ^(a) ^	–	–	2 (50.0)	2 (11.8) ^(a) ^
	Relatives	11 (34.4)	–	5 (100.0)	–	2 (50.0)	4 (23.5)
	Public stakeholders ^(b) (c) ^	15 (46.9)	–	–	4 (100.0) ^(b) ^	–	11 (64.7) ^(c) ^
Sex, female (N = 32)		23 (71.9)	3 (100.0)	4 (80.0)	2 (50.0)	2 (50.0)	13 (76.5)
Age in years (N = 31)		37.0 – 85.0	51.0 – 74.0	37.0 – 72.0	42.0 – 81.0	59.0 – 79.0	49.0 – 85.0
		66.3±12.2	66.0±13.0	54.3±14.4	62.5±16.1	64.8±9.5	69.5±10.7
Number of children (N = 32)		0 – 4	2 – 3	0 – 2	0 – 3	0 – 4	0 – 4
		1.7±1.2	2.3±0.6	1.0±1.0	2.0±1.4	1.3±1.9	1.8±1.0
Migration background (N = 32)		1 (3.1)	–	–	–	–	1 (5.9)
Marital status (N = 32)	Single	2 (6.3)	–	–	1 (25.0)	–	1 (5.9)
	Married/Civil partnership	20 (62.5)	1 (33.3)	5 (100.0)	2 (50.0)	4 (100.0)	8 (47.1)
	Divorced	4 (12.5)	2 (66.7)	–	–	–	3 (17.7)
	Widowed	6 (18.8)	–	–	1 (25.0)	–	5 (29.4)
Final school degree (N = 32)	Lower degree	8 (25.0)	–	1 (20.0)	1 (25.0)	–	6 (35.3)
	Average degree	14 (43.8)	2 (66.7)	3 (60.0)	1 (25.0)	2 (50.0)	7 (41.2)
	Higher degree	10 (31.3)	1 (33.3)	1 (20.0)	2 (50.0)	2 (50.0)	4 (23.5)
Education (N = 32) ^(d) ^	Vocational (school or company) degree	17 (53.1)	2 (66.6)	3 (60.0)	2 (50.0)	–	11 (64.7)
	Technical/master (school or academy)	5 (15.6)	–	1 (20.0)	1 (25.0)	–	3 (17.6)
	University or college degree	9 (28.1)	1 (33.3)	1 (20.0)	1 (25.0)	4 (100.0)	2 (11.8)
	Other (e.g., technician)	1 (3.1)	–	–	–	–	1 (5.9)
Occupation (N = 32) ^(d) (e) ^	Professional	4 (12.5)	1 (33.3)	–	–	2 (50.0)	1 (5.9)
	Technician/Associate professional	4 (12.5)	–	2 (40.0)	1 (25.0)	–	1 (5.9)
	Worker (e.g., services and sales)	24 (75.0)	2 (66.7)	3 (60.0)	3 (75.0)	2 (50.0)	15 (88.2)
Previous profession (N = 32) ^(d) (e) ^	Manager	9 (28.1)	–	1 (20.0)	1 (25.0)	3 (75.0)	4 (23.5)
	Professional	2 (6.3)	1 (33.3)	1 (20.0)	–	–	–
	Technician/Associate professional	4 (12.5)	–	2 (40.0)	1 (25.0)	–	1 (5.9)
	Worker (e.g., services and sales)	9 (28.1)	–	1 (20.0)	–	–	8 (47.1)
	Elementary occupation	1 (3.1)	–	–	1 (25.0)	–	–
	Other (e.g., housewife or home care)	7 (21.9)	2 (66.7)	–	1 (25.0)	1 (25.0)	4 (23.5)
Current profession (N = 32) ^(d) (e) ^	Manager	1 (3.1)	–	–	–	–	1 (5.9)
	Professional	1 (3.1)	–	1 (20.0)	–	–	–
	Technician/Associate professional	3 (9.4)	–	1 (20.0)	1 (25.0)	–	1 (5.9)
	Worker (e.g., services and sales)	2 (6.3)	–	1 (20.0)	–	–	1 (5.9)
	Pension/Early retirement	22 (68.8)	3 (100.0)	2 (40.0)	2 (50.0)	3 (75.0)	13 (76.5)
	Other (e.g., housewife or home care)	3 (9.4)	–	–	1 (25.0)	1 (25.0)	1 (5.9)

N, number of participants; M, mean; SD, standard deviation; MMSE, Mini-Mental State Examination; PlwD, people living with dementia. ^(a) ^One participant participated in both an individual interview and a focus group. ^(b) ^One public stakeholder experienced dementia-related symptoms (e.g., forgetfulness and loss of orientation). ^(c) ^Three participants participated as public stakeholders but were also relatives of people with neurodegenerative diseases (2 dementia and 1 other disease). ^(d) ^The categories are grouped according to the International Standard Classification of Occupations (ISCO-08) (Available from: https://www.ilo.org/public/english/bureau/stat/isco/isco08/)/. ^(e) ^Multiple answers are possible.

The individual and dyadic interviews lasted from 18 to 46 minutes (mean 30 minutes), and the focus group interviews lasted from 64 to 75 minutes (mean 69 minutes). Women (*n* = 23) comprised the majority of the participants (N = 32). The overall mean was 66 years (N = 31) with a standard deviation of 12.2 years. The participants had a mean of 1.7 children (N = 32), while the PlwD (N = 6) had a mean of 1.5 children. Most participants were married or lived in civil partnerships, had an average school degree with a vocational degree, were workers in services, sales, or management, and received a pension or were retired. One person had a migration background. The educational degree of the PlwD (females and males, each *n* = 3) was a vocational (school or company) degree (*n* = 3) and a university or college degree (*n* = 3). Four PlwD provided a communicated verified diagnosis of early dementia, and two PlwD provided a documented diagnosis of dementia. Three of the six PlwD agreed to participate in a voluntary MMSE and had MMSE scores ranging from 10 to 30, with a mean score of 22.0 and a standard deviation of 10.6.

The feedback meeting (N = 15) lasted for 60 minutes. The participants (*n* = 12 females and *n* = 3 males) that were present participated in all types of interviews as follows: PlwD (N = 3), relatives (N = 6), and public stakeholders (N = 6).

### 2.4 Data collection

Two researchers (SK and SB) conducted the individual interviews, dyadic interviews, and focus group interviews between August 2018 and April 2019. Due to the different data collection methods (triangulation), we aimed to obtain a reliable picture of emotional safety in the context of dementia. Semi-structured guided individual face-to-face interviews were conducted with each target group. If requested by the PlwD and their relatives, we conducted dyadic interviews, which have been shown to provide support and strengthen feelings of safety [18]. The semi-structured guided focus groups were based on a scoping focus group design with an ”individualistic social psychology perspective” [19] to consider the individual view of each participant. In the feedback group, communicative validation of the results was performed, enabling a meta-perspective of all results of all participant groups. Two moderators (SK and SB) presented the generated main and subdimensions to the participants for verification or adjustment as necessary.

All interviews and focus groups were audio-recorded and observed with field notes recorded by another research team member [20]. The interviews were transcribed by an external transcription office according to published guidelines [21, 22]. According to Pesonen and colleagues (2011), the presence of a third party (who was not personally interviewed) was allowed in selected interviews if the participant expressed a desire for support. To ensure ongoing consent, the participants were asked to provide their consent to participate before each interview. The interviews started with the researcher providing an introduction and explaining the rationale and aim of the interview. In general, only a few questions guided the interviews, and communication techniques, such as paraphrasing and requests to narrate, were applied to gain as much information as possible regarding the themes. After the interviews, the PlwD were asked to voluntarily complete the MMSE [23]. Structural data (e.g., concerning age and education) were collected separately by a structural questionnaire before the interviews by the team from the non-statutory welfare association. This team collected participatory data by recording field notes using a predefined form during their meetings.

The participants were free to choose the time and location of the data collection [17]. The interviews occurred during the day in a room familiar to the participants, the project buildings, other public meeting places, the participants’ home, or the participants’ workplace.

To support the group of PlwD during the focus group interviews and avoid opinion leadership, the introduction question was posed to all participants separately. Each participant had the opportunity to present his or her opinion. To support the participants, especially the people with cognitive impairments, during the interviews, cards were used as stimulus materials [19] and, in the case of dementia, as a re-minder. The cards contained the following topics related to emotional safety, which could be ide-ntified from previously collected data and a systematic review [1]: “everyday tasks”, “places”, “aids/ technology”, “personal contacts”, “leisure time (e.g., trips, holidays, and sports)”, “work”, “home”, “traffic”, and “dementia diagnosis”. Furthermore, a card with a free text field was included. The cards were multiplied by four and laid in a structure manner in front of the participants on each side of the table. The participants were free to choose a number of cards with their preferred topics. The selection could be changed during the course of the interview or reference could be made to new cards. The participants were informed that they could discuss with each other.

### 2.5 Interview guides

The interview guides (see Supplementary Tables 1 and 2) were developed following the “SPSS method”, which includes four steps, namely, “(S) collecting questions in relation to the phenomenon of interest”, “(P) checking against the state of research and openness”, “(S) sorting in relation to the sequence and/or content”, and “(S) subsuming topics in relation to prompts, probes and key-words” (translation SK) [24]; these steps were supervised by the research team and pretested. All guided interviews were subdivided into the following three parts: introduction, main part, and closing. The introduction (individual and dyadic interviews) contained an initial question regarding a usual day in the interviewees’ everyday lives and a following question regarding non-daily activities. Following the previously mentioned activities, the next question addressed the feeling of safety/lack of safety in the mentioned situations. The second question asked the participants what is needed to feel safe. The closing part included a question regarding whether the interviewees wanted to discuss anything else related to the feeling of safety.

The introduction to the focus groups started with a warm-up question regarding interest in the topic of feeling safe in the context of dementia. The main part allowed an open discussion regarding feeling safe and a second question regarding what would be recommended to enable a feeling of safety. At the end, each participant was asked whether he or she had anything else important related to the feeling of safety to share.

### 2.6 Data analysis and synthesis

The data analysis was based on a predominately inductive content analysis approach [25] using MAXQDA 11 (VERBI Software, 2014). Inductive analyses allow open coding and the development of categories on different data levels. Additionally, deductive subcategories were guided by the definition of emotional safety and the research question; these subcategories included “needs”, “conditions”, “strategies”, “influencing factors”, and “constructs related to emotional safety”.

First, all data of each transcript were examined. The analysis of the transcript sections was performed according to our research question. In each target group, we analyzed the participants’ explicit statements referring to emotional safety and implicit statements that were not directly related to emotional safety but described in the interview to avoid conformation bias and identify phenomena related to emotional safety in the context of dementia. Second, the categories were developed and concretized during repeated analyses of these sections separately. Third, the categories were analyzed according to the five deductive subcategories described above. In the final step, we compared and analyzed the content of the subcategories among the categories and among the perspectives. We analyzed the data from the interviews (SB, SK and CB) and participatory approach (FW and SB) separately. All interviews were initially coded by one coder (SB). Two senior researchers supervised the coding procedures (SK), checked all coding (SK and CB), and provided coding recommendations (SK and CB). The data synthesis (SK and SB) included an analysis of the categories while considering the different perspectives of the participants, an analysis of the common and varying dimensions and an analysis of the contradictory, complementary and interrelating findings. The analysis team (SK, SB, and CB) discussed the categories and data synthesis and made adaptations in case of need.

### 2.7 Ethical approval

The EMSIDE model project was approved by the ethics committee of the German Society of Nursing Science (project reference number: 17-013).

## 3 RESULTS

The phenomenon of the need for emotional safety in the context of dementia was explicitly mentioned by each target group. The experience of feeling safe was described by relatives as “not being uncertain and not being afraid” (ID 39, 41, 42). A relative described that people in general who are afraid search for safety (ID 39). Additionally, PlwD described the feeling of being safe in the context of being afraid (ID 04). The participants also differentiated between something that someone likes and the feeling of safety (ID 01). Emotional safety was described as a human need and was relevant to all participant groups (ID 02, 03, 21, 24, 39, F2, FB). PlwD and relatives described emotional safety as a human need that is of high relevance (ID 03, 24, 39).

“I need the feeling of structure and safety.”

(ID 03, PlwD)

The needs of the PlwD were related to staying independent (ID 40), having structure in daily life (ID 03), and staying cognitively fit (ID F1). According to the relatives, there is a need for normality (ID 24, 40, F1), staying in familiar surroundings (ID 24, 37, 38, 41, 42, FB), performing daily activities (ID 37, 38) and common undertakings (ID F3). The relatives described the wish to do things correctly when interacting with each other (ID 02). The public stakeholders described that relatives have the greatest need for emotional safety (ID 21). The search for help can be an expression of this need (ID 21).

“For relatives themselves, safety is the most important thing, a reason to take action at all and why they seek help at all.” (ID 21, public stakeholder)

### 3.1 Dimensions related to emotional safety

In total, five clusters of dimensions, 16 main dimensions and 373 subdimensions were developed. We were able to show that the conditions, strategies, and influencing factors (related to the diagnosis of dementia and psychosocial, biographical, physical, and economical factors) were allocated to the following five clusters: “social togetherness”, “personal condition”, “health”, “physical environment”, and “society”. Emotional safety in the context of dementia was described by all participant groups in relation to at least one other construct (e.g., well-being). The clusters comprise the 16 main dimensions as follows:

(i) “social togetherness” (“perceived communality”, “social interaction”, “support”, and “use of dementia services”);
(ii) “personal condition” (“feeling of structure”, “perceived familiarity”, “home as a space for safety, and for personal preferences”);
(iii) “health” (“perceived changes”, “process of dia-gnosis”, “communication of diagnosis”, and “resilience (coping)”);
(iv) “physical environment” (“mobility” and “sa-fety”);
(v) “society” (“normality of the topic of dementia” and “fundamental awareness”).

The results related to the main dimensions were classified based on the following deductively defined subcategories: a) “conditions and strategies” (Table 2), b) “influencing factors” (Table 3), and c) “constructs related to emotional safety”.

**Table 2:** Dimensions of emotional safety: conditions and strategies (explicit statements)

Cluster of dimensions	Main dimension				Subdimensions
			(Individual/dyadic interviews^*^)		(Individual interviews)	(Focus group interviews)
			PlwD (N = 5) and relatives (N = 2)	Relatives (N = 5)	Public stakeholders (N = 4)	All perspectives (N = 17)
Social togetherness	Social interaction	CO	/	•Positive attitude (acceptance of	•Positive attitudes (open-mindedness,	•Positive attitude of others
				PlwD, not seeing PlwD as sick)	not seeing PlwD as sick)	(acceptance of PlwD)
(*n* = 101)	(*n* = 35)			•Sympathy	•Sympathy	•Appropriate communication and behavior
				•Knowledge, experience, information,	•Experience/knowledge	(body language, eye contact) of others
				learning (about PlwD behavior	•Emotional safety of others	•Continuous learning
				and tasks of daily living)	(ID 22, 23, 40)	•Knowledge/information (symptoms,
				•Competence in empathy		course of the disease,
				•Emotional safety of others		dementia-related behavior)
				(ID 02, 25, 26)		•Assurance of correctly handling
						risk situations
						(F1 – F3, FB)
		ST	/	•Education for relatives and public	•Education (appropriate behavior	•Trainings for relatives and public
				stakeholders related to dealing with	dealing with PlwD by participating	stakeholders (selecting needed information)
				stressful situations, awareness, sympathy,	meetings)	•Significant others as intermediaries
				and understanding challenging behavior	•Reflecting own behavior	•Relationship building (taking time)
				•Relationship building (starting with the	•Appropriate communication (not	•Become familiar with PlwD
				first contact)	addressing impairments, give a plan,	•Contact with people who are good for PlwD
				•Provide PlwD a feeling of being accepted	establish contact with reality,	•Increase situational awareness
				•Visits considering the needs of PlwD	create a feeling that everything is “good”)	(adaption of physical conditions)
				•Act empathetically	•Provide a feeling of being	•Paying attention/adaption (outer
				•Using recommendations for action	accepted for PlwD	appearances, communication)
				(ID 02, 24 – 26)	(ID 21 – 23)	•Working, using feelings
						•Mirroring/following PlwD behavior
						•Challenging behavior: divert by visual
						signs
						•Creating calm atmosphere
						(F1 – F3, FB)
	Perceived	CO	•Experience of congruencies	•Bodily contact	•Living in relations	•Bodily contact
	communality		•Taking pleasure in positive	•Feeling of togetherness	•Closeness	•Feeling of togetherness
	(*n* = 28)		mood of relatives	•Closeness to significant others	(ID 21, 22)	•Closeness to significant others
			•Close relationship^*^	(ID 02, 24 – 26)		(F3, FB)
			•Get on well with someone^*^
			(ID 01, 37, 38, 40– 42)
		ST	•Common activities^*^	•PlwD contact	•Common activities	•Activities in familiar surroundings
			•Common realization of wishes	•Presence of family	•Situational interaction	•Involvement of significant others
			•Taking care (of each other)	•Experience of community (friends)	•PlwD contact	•Interaction with like-minded people (relatives)
			(ID 01, 37, 38, 41, 42)	•External planning support	•Taking time (to listen)	•Taking care (of each other)
				•Changing homes	(ID 21, 22)	(F1 - F3)
				(ID 02, 24– 26, 39)
	Support	CO	Willingness to get support	•Additional support	•Willingness of PlwD to get support	•Assurance to be well cared for
	(*n* = 27)		(ID 03)	•Ability to plan safe conditions	•Trust in others	•Continued access for support
				•Assurance to (quickly) get additional	•Appropriate behavior of the	•Knowledge of where to get help
				support	person proving support	(F1 – F3, FB)
				•Significant others living in immediate	•Need-oriented services
				environment	(ID 21, 23)
				(ID 02, 24, 26, 39)
		ST	•Assistance in daily situations	•Developing networks	•Education (understanding warning	•Contacting own network
			•Cognitive assistance	•Participation in information events	signals, disease)	•Common activities with significant others
			•Situational creation	•Stimulating capacity building	•Involving relatives, friends,	•Behavioral guidance considering individual needs
			(mobile phone and telephone	•Knowing assurance is of importance	neighborhood and professionals	•Transmission by first contact person
			number of road assistance)^*^	for PlwD	•Task allocation considering	•Improving safety interventions in
			(ID 03, 37, 38)	(ID 02, 24)	competences and needs of PlwD	unknown areas
					(ID 21)	(F2, FB)
	Use of	CO	•Dementia need-oriented	•Need-oriented services	•Assurance: daily routines, guarantee
	dementia		group offers^*^	(for PlwD and relatives)	of care continuity	/
	services		•Mobility services and aids	•Residential services	•External support for everyday tasks	•Offers for PlwD and/or relatives
	(*n* = 11)		(electric mobility scooters) ^*^	(ID 02, 24, 26)	24-hour care	(considering information, communication,
			(ID 37, 38, 41, 42)	•Offers for PlwD and/or relatives		interaction, activities, training,
		ST	/	(activities for several generations,		therapy, support)
				information, general support,	•Appropriate communication	(F1, F2, FB)
				support in daily life activities,	from professionals
				e.g., grocery shopping	(ID 21)
				and public transport)
				•Care (medical, daily living)
				(ID 02, 24, 26, 39)
Personal	Feeling	CO	Knowledge about	•Routines (ID 25)	•Routines (ID 21, 22)	•Counting on something
condition	of structure		daily competences, wishes			•Perceived control
(*n* = 61)	(*n* = 20)		and experiences			•Knowledge about safety in structures
			(ID 01)			(F1, FB)
		ST	In daily living: adhere	•Relocation in settings with	•Routines (create artificial safety)	•Routines/requesting daily activities
			to existing structures,	consistent structures	•Enforce routines when others get lost	•In case of changes, learning new structures
			create structures	•Identification of structures and	•Enable routines	•Creating structures
			(ID 01, 03)	adherence to it	(ID 21, 22)	•Good timing of planned activities
				(ID 25)	•Adaption of structures (flexible, individual)
						•Planning daily activities
						(F1, FB)
	Home as	CO	•Knowing and safeguarding	•Familiarity (ID 26)	/	•Protection against confrontation with disease
	a space		basic human needs	•Protection from physical conditions
	for safety		•Protection (health risk			•Protection against unsafe behavior
	(*n* = 18)		situations, burglaries)^*^			(F1 – F3, FB)
			(ID 37, 38)
		ST	•Staying at home (days	•Staying at home	/	•Staying at home
			not feeling well)	•Relocation in case of loneliness		•Being visited by others
			•Doors locked at night^*^	•Living community		•Night care
			•Avoidance (communication	•Build confidence		•Day care/day care unit
			and thinking on bad days)	(ID 24 – 26)		(F1 – F3)
			•Be alone
			(ID 03, 37, 38)
	Perceived	CO	Environment (physical/social)^*^	•Environment (physical, social)	•Routines in familiar	•Environment (physical, social)
	familiarity		(ID 37, 38, 41, 42)	•Familiar preferred activities	environments (ID 22)	•Routines
	(*n* = 18)			•Knowledge (positive behavior		(F1 – F3, FB)
				of PlwD and others in contact)
				(ID 02, 24 – 26, 39)
		ST	Regular contacts^*^	•Consistent structures	•Staying in safe settings	•Staying at home
			(ID 41, 42)	•Confidence building in	•Physical adaptions	•Avoiding (unknown localities or persons,
				different settings	(ID 22)	distant relatives, huge groups)
				•To leave familiar things,		(F2, F3)
				PlwD in own settings
				•Familiar activities
				•Relocation in settings with less
				fluctuation
				(ID 02, 24 – 26)
	Home as	CO	•Opportunity to live according	/	/	•Things connected with emotions
	a space for		to own preferences^*^ (ID 37,38)			•Private space
	personal		•Time flexibility to suit own			(F1, FB)
	preferences		preferences^*^ (ID 37, 38)
	(*n* = 5)		•Not changing homes	/	/	/
		ST	(ID 37, 38)
Health	Perceived	CO	•Personal attitudes (reduced	•Perception of fully functional	/	/
(*n* = 48)	changes		expectations of oneself,	human sense (ID 25)
	(*n* = 30)		increased trust in others)	•Finding flexible and situational	•Physical environment adaption	•Visual aids (fixed contact information
			•Increased trust in God	strategies	•Avoidance	(note, card), familiar signs, landmarks)
			(ID 03)
		ST	•Mobilizing resources (personal,	•Staying at home	•Providing structures	•Approaching PlwD to take a taxi
			technical, cognitive)	•Support in situations of	•Hiding impairments	(in case of orientation loss)
			•Reflection (of uncertainties	symptom confrontation	•Requesting needs	•Commonly doing tasks
			in everyday life)	•Bonding (emotionality/empathy)	•Finding need-oriented support	•Request of external advice
			•Finding alternatives for action	•Adapting structures by considering	•Working with memories	(F2, F3)
			•Task allocations related	the needs of PlwD (in health care)	•Appropriate communication
			to current skills	•Aids in public space (GPS)	(ID 21, 22)
			•Constant physical environment	(ID 02, 24 – 26)
			•Planning situations
			•Getting support (in situations
			with reduced self-efficacy,
			in potentially hazardous situations)
			•Avoidance
			•Hide behind the disease
			(ID 01, 03, 40)
	Communication	CO	Unknowingness of others of the	Knowledge about diagnosis (ID 24)	/	/
	of diagnosis	ST	diagnosis of dementia (ID 04)	•Masking	/	•Openness (symptoms, diagnosis)
	(*n* = 7)		Openness (ID 03, 04)	•Openness		•Information sign/card “have dementia”
				(ID 02, 24)		(F1 – F3, FB)
	Process of	CO	/	/	/	•Definite diagnosis (to try to act in small steps)
	diagnosis	ST	/	/	/	•Contact with competent professionals
	(*n* = 6)					(F1, F3)
						•Self-diagnosis
						•Politics
						•Time for diagnosis by physicians
						•Dementia-specific education for
						professionals
						(F1 – F3)
	Resilience	CO	•Developing new self-esteem	/	/	•Acceptance of changes (FB)
	(coping)	ST	Using diagnosis for own needs and	/	Relatives repress perceived changes	•Learning to deal with the disease (ID FB)
	(= 5)		resources (ID 03)		(ID 21)
Physical	Mobility	CO	/	/	/	/
environment	(*n* = 11)	ST	•Finding alternatives for action	/	•Staying at home	•Visual landmarks
(*n* = 16)			•Being careful		•Care	•Avoiding driving cars
			•Increase concentration		(ID 21,22)	(F2)
			•Avoidance (public spaces) ^*^
			•Driving in minor traffic^*^
			•Learning to drive scout mobile^*^
			•Living on the ground floor^*^
			(ID 03, 37, 38)
	Safety	CO	/	/	/	/
	(*n* = 5)	ST	Using (learned safety behavior,	/	•Lower blinds at dusk	•Technical aids internal and external
			physical safety aids) ^*^		•Turn on light	(monitoring, emergency)
			(ID 37, 38)		(ID 22)	•Adaption of physical environment
Society	Normality	CO	/	•Open communication	/	(considering abilities, avoiding risks)
(*n* = 10)	of the topic			•Presence of topic of dementia		(F1 – F3)
	of dementia	ST	/	in society	/	•Positive attitudes (FB)
	(*n* = 8)			(ID 24)		•Education (society, starting with children)
				•Public relations for dementia services		•Professional presentation of the topic
				•Creating a new picture of		of dementia
				dementia		(F2, FB)
				(ID 02, 24)		•Presence of the topic
	Fundamental	CO	/	/	•Awareness of competences (ID 23)	/
	awareness		/	/	/
	(*n* = 2)	ST			•Education (ID 23)

^*^Dyadic interviews; CO, Condition; ST, Strategy; PlwD, People living with dementia.

**Table 3:** Dimensions of emotional safety: influencing factors (explicit statements)

Cluster	Main dimensions			Subdimensions
		(Individual/ dyadic interviews)		(Individual interviews)	(Focus group interviews)
		PlwD (N = 5) & relatives (N = 2)	Relatives (N = 5)	Public stakeholders (N = 4)	All perspectives (N = 17)
Social togetherness	Social interaction	•Knowledge (about dementia/	•Symptom awareness of PlwD	•Fast pace of society	•Characteristics of PlwD
		diagnosis)	•Stage of dementia	•PlwD; restricted capacity	•Stage of dementia
(*n* = 68)	(*n* = 24)	•Lacking awareness of changes	•Knowledge (about dementia	to understand fears for no reason	•Inappropriate behavior or clothes
		•Fear of negative reactions	symptoms/diagnosis)	•Lacking knowledge (about correct	•Sensible awareness in contact
		of others	•Sensitivity and emotionality of PlwD	behavior)	•Uncertainty in interaction/behavior (PlwD)
		•Confrontation with symptoms	•Inappropriate behavior of others	•Negative attitude (communication,	•Lacking knowledge
		(ID 03, 04, 40)	•Difficulty in sympathy/patience	behavior)	•Challenging behavior cannot
			•Negative picture of dementia	(ID 21 – 23, 40)	always be avoided
			•Practicability of empathy		•Perceived familiarity
			(ID 02, 24 – 26)		(F1 – F3, FB)
	Perceived	Barriers for experiencing	•Characteristics of PlwD	•Practicability of maintaining presence	•Characteristics of PlwD
	communality	togetherness (physical and	•Stage of dementia	•Awareness in contact with PlwD	•Stage of dementia
	(*n* = 16)	psychosocial) (ID 40)	•Common history	•Presence of significant others	•Role/relationship
			•Loss of partner/loneliness	(ID 22)	•Fear of activities
			•Home setting		•Physical environment
			•Employment of the family		•Environmental changes
			(ID 02, 24, 26, 39)		(F2, F3)
	Support	•Perceived health condition	•Stage of dementia	•Characteristics of PlwD	•Challenging behavior of PlwD
	(*n* = 16)	•Parental upbringing	•Inter-role conflicts	•Rejection of support	•Perceived anonymity
		•Experience with own parents	•Knowledge (about dementia diagnosis)	•Inter-role conflicts	•Lack of support
		(ID 03, 40)	•Health of significant others	•Impaired family resources	(F2)
			•Physical distance	•Physical distance
			(ID 02, 24, 26, 39)	(ID 21)
	Use of	/	•Stage of dementia	•Characteristics of PlwD and other	•Barriers of coping (lack of
	dementia		•Barrier of lacking support, not finding	•Difficult estimation: situation of PlwD	acceptance of diagnosis)
	services		or perceiving information	•Family individuality	•Time since diagnosis
	(*n* = 12)		•Health condition of significant others	•Rejection of care of PlwD	•Stigmatization
			•Not fitting dementia services^*^	•Limited resilience	(F1, F2)
			(ID 02, 24, 26)	(ID 21)
Health	Perceived	•Confrontation with impairments	•Confrontation: (intensified) symptoms	•(Conscious) dementia-related changes/	/
(*n* = 27)	changes	•High risk consciousness	•Symptom awareness of PlwD	confrontation in daily living
	(*n* = 14)	•Symptoms of dementia	•Lack of support	•Loss of former live
		•Interplay of psychosocial and	•Loss of safety structures	•Symptoms of dementia
		dementia-related changes	•Difficulty for others: symptom	•Individuality Knowledge relatives:
		(ID 01, 03, 40)	recognition	PlwD behavior
			(ID 02, 25)	(ID 21, 22)
	Communication	Barriers of communication	•Notable decline in contact behavior	/	•Lack of realization of having dementia
	of diagnosis	(own attitude and attitude	•Openness when others notice symptoms		•Observations of symptoms by others
	(*n* = 7)	of others)	•Guardedness if competences exist		•High sensibility: negative reaction from
		(ID 04, 40)	(ID 02, 24)
	Process of				others
	diagnosis	/	/	/	(F1, F2)
	(*n* = 6)				•Barriers (roles, behavior, picture of
				dementia in society and knowledge of dementia)
					•Biological fitness
					•Uncertainty about dementia changes
					•Different advice
					•Perceived negative reactions from physicians
					regarding own observations
					•Impaired diagnosis process
					of dementia
					(F1 – F3)
	Resilience	/	/	/	/
	(coping)
	(*n* = 0)
Personal	Feeling of	•Negative reactions of others	•Characteristics of PlwD	•Loss of control	•Characteristics of PlwD: external structures
condition	structure	when keeping daily routines	PlwD to become used to new structures	•Loss of competences	are not fitting to own structures
(*n* = 27)	(*n* = 10)	•Lack of physical structure	•Loss of significant others	to plan	•Knowledge of risks, not be able
		(ID 03)	•Difficulty keeping daily	(ID 21)	to keep structure
			routines at home		(FB)
			(ID 25)
	Perceived	Perceived unfamiliar	•Stage of dementia	Confrontation with unknown	•Stage of dementia
	familiarity	environment^*^	•Characteristics and experiences of PlwD	cultures	(F2)
	(*n* = 8)	(ID 37, 38)	•Time-consuming development of trust	(ID 22)
			in new people and localities
			•Loss of significant others
			•Unfamiliar environment
			(ID 02, 25, 26, 39)
	Home as a	•Sorrow leaving home	•PlwD leaving home	/	•Perceived changes
	space for	•Physical environment^*^	•Expected loneliness of PlwD	•Characteristics of PlwD
	safety (*n* = 8)	•Perception of bad days with	(ID 24, 26)		•Physical environment
		increased symptoms			(F1 – F2)
		(ID 03, 38, 40)
	Home as a		/	/	/
	space for
	personal	Not fitting external dementia
	preferences	services^*^
	(*n* = 1)	(ID 37, 38)
Physical	Mobility	•Perceived changes/ self-efficacy	/	Darkness (ID 22)	•Stage of dementia
environment	(*n* = 7)	•Health status^*^	•Physical impairments
(*n* = 11)		•Rural areas^*^			•Lack of technical solutions for PlwD
		(ID 03, 37, 38)			(F2, F3, FB)
	Safety (*n* = 4)	Health-/risk situations^*^	/	Darkness (ID 22)	•No safety although using facilities
		(ID 03, 37, 38)			•Night
					(F3)
Society	Normality of	/	View of dementia in society,	/	•Stigmatization
(*n* = 4)	the topic of		e.g., negative		•Knowledge about diagnosis by others
	dementia (*n* = 4)		(ID 02)		•Changed society
					(F2, FB)
	Fundamental	/		/	/
	awareness
	(*n* = 0)

^*^Dyadic interviews; PlwD, People living with dementia.

The comparison of the explicit and implicit data that addressed the topic of emotional safety from the interviews and participative data showed no new main dimensions. Additionally, the data from the feedback group showed no new main dimensions. However, the feedback group substantially contributed by reflecting upon all perspectives of the participant groups on a meta-level and in the development of the overarching dimension “living and learning in relations”. However, it cannot be ruled out that further single subdimensions could be developed based on further interview data depending on the individual participants or other settings.

The overarching dimension resulting from all explicit data and feedback data, i.e., “living and learning in relations”, comprises the following two aspects: 1) the interaction of people in the context of dementia and people’s relation to the topic of dementia, and 2) the (common) learning of all participants. All developed clusters contained the dimensions of 1) interaction of people in daily life. Furthermore, all clusters contained the terms 2) “learning”, “education”, “training”, “competences”, “knowledge”, or “experiences”. These two core aspects in the context of dementia, i.e., “living and learning in relations”, were emphasized as the most important aspects in the feedback group. Additionally, the individual needs of each person and individuality of his or her situation were central in the feedback group. For example, the participants of the feedback group stressed that structure also needs a degree of flexibility and should be provided by considering and understanding individual needs.

To develop solutions and strengthen the feeling of safety, one participant suggested that individuals need to cooperate and develop a situational need- and resource-oriented “emotional safety thinking” (F2). Another participant suggested that “good togetherness” can provide emotional safety to all individuals (FB) as the feeling of safety of one person (e.g., formal caregiver) can affect the feeling of safety of another person (e.g., PlwD) (FB).

### 3.2 Conditions and strategies of emotional safety in the context of dementia

In total, 236 subdimensions of conditions and strategies were developed (Table 2). In all five clusters, we observed several common, complementary, contradictory, and varying conditions and strategies from a multiperspective point of view. Not all main dimensions were addressed by all participant groups. We also observed that the content was interrelated.

### 3.3 Common conditions and strategies of the participants and content interrelations

The cluster “social togetherness” contained the most frequently mentioned conditions and strategies (*n* = 101) of all clusters and was addressed by all participant groups. The main dimensions “perceived communality” (*n* = 28), “support” (*n* = 27), and “use of dementia services” (*n* = 11) were addressed by each group of participants. The main dimension “social interaction” (*n* = 35) was among the largest main dimensions containing most of the categories. Although there were no subcategories based on the (dyadic) interviews with PlwD in this dimension, both the conditions and strategies of the other dimensions contained aspects related to learning as a com-monality. The conditions were related to needed “knowledge”, e.g., regarding symptoms, course of the disease, dementia-related behavior, and “experience”. Other common conditions were related to personal traits (e.g., positive attitudes) and communicative, psychosocial and professional skills (e.g., empathy and consideration of appropriate communication and behavior). A dimension of “continuous learning” as a condition could be developed based on the focus group data. Related to such conditions, we identified strategies containing “education” and “training”, e.g., “addressing stressful situations, awareness, sympathy, and understanding challenging behavior” and/or “appropriate behavior addressing PlwD by participating in meetings” and/or the creation of a need-oriented environment for relationship building. Further strategies related to “social interaction” addressed appropriate communication and performance regarding “relationship building” and the creation of “(...) a feeling of being accepted” among PlwD. In this context, the relatives and public stakeholders assumed that the emotional safety of others, e.g., of the relative, is also a condition for one’s own emotional safety, e.g., of the PlwD.

“*If, (...) I - am trained or if I know how to deal with it, (...) then I can resonate safety and can also show the sick person, I am safe. (...)” (ID 02, relative,* “*social interaction”)*

Additionally, the other clusters contained aspects of learning as follows: the cluster “personal condition” contained “knowing and safeguarding basic human needs”; the cluster “health” contained “learning to cope with disease”; the cluster “physical en-vironment” contained “using learned safety behavior and physical safety aids”; and the cluster “society” contained “education (society, starting with children)”. Specifically, in the feedback group, “learning” was the core condition emphasized and was chosen as the most important by all participants.

In the cluster “social togetherness”, another large main dimension identified was “perceived communality”, which covered all perspectives of the target groups. The conditions were predominantly related to relationship and/or relations, e.g., “feeling of to-getherness”, “close relationships” and “living in relations”. Here, the strategies were also interrelated with the conditions as expressed by “PlwD contact”, “common activities”, and/or “taking care (of each other)”. Additionally, this aspect was discussed in the feedback group and appeared to be chosen as a core dimension of the results. It was stressed that “living in relations” extends beyond relationship. Importantly, the “feeling of togetherness” is a condition of emotional safety, and the perspective of society regarding the topic of dementia considering not only “knowledge” but also “acceptance” and “respect” was stressed in terms of “living in relations”. Although the cluster “society” contained fewer categories and not all individual perspectives were addressed, we identified “education” in the perspective of the public stakeholders and focus groups. The strategies included public relations, “professional presentation of the topic of dementia” and “education of society (starting with children)” with the aim to learn about the topic of dementia in a manner that shows the positive sides. The main dimension “fundamental awareness” (*n* = 2) included one condition related to public competences that can be improved through education. In this context, the main dimension “normality of the topic of dementia” (*n* = 8) was stressed by relatives in terms of “open communication” and the “presence of the topic of dementia in society”.

*“(...) if - people deal with it in a more normal way, they [relatives] also feel safer.” (F2,* “*normality of the topic of dementia”)*

Furthermore, “support” was another dimension addressed in all perspectives. From the perspective of PlwD and public stakeholders, “support”, as a main dimension, includes the willingness of PlwD to obtain support and related factors (e.g., trust). The relatives and focus group participants mainly referred to conditions of care and daily routines (e.g., assurance to (quickly) obtain additional support and be well cared for and knowledge of where to receive help). The strategies ranged from need-oriented assistance in daily life (e.g., personal, technical and cognitive) to the development of a support network. The “use of dementia services” as a main dimension is need-oriented and contains strategies related to information, communication, interaction, activities, training, therapy, and support for PlwD and/or relatives.

Additionally, both the clusters “personal condition” and “health”, which also contained nearly half of the categories, were addressed by all participant groups. The following three main dimensions of the two clusters were addressed by all participant groups: “feeling of structure” (*n* = 20), “perceived familiarity” (*n* = 18), and perceived changes (*n* = 30). The main dimension “feeling of structure” comprised knowledge and awareness of past and future structures (e.g., routines).

“*I feel safe there. And, I can also dawdle, and I can also dream, but I like to have a structure where I know I have done this. I have done this now. I have done that. I have been able to do that. I wanted to do that (...)” ID 01, PlwD,* “*feeling of structure”)*

The strategies were related to the identification and creation of supporting circumstances for existing structures and the implementation of new structures. Structures and knowledge allow safety. Additionally, the feedback group emphasized that a “feeling of structure” also should allow flexibility, e.g., rigid time structure, in terms of individual needs. Additionally, “learning [to address] new structures” was highly important. The conditions of the dimension “perceived familiarity” were related to the physical and psychosocial environment and structure in everyday life. All strategies concentrated on maintaining this familiarity (e.g., through regular contacts).

Many conditions and strategies mentioned in the different participant groups can be considered complementary; for example, in the main dimension “safety” (*n* = 5) (cluster “physical environment”), the “technical aids internal and external (monitoring, emergency)” and “adaption of physical environment (considering abilities, avoiding risks)” can be considered complementary. However, there were also intrapersonal complementary conditions. For example, in the cluster “health”, from the perspective of the PlwD, the dimension “resilience (coping)” (*n* = 5) included the complementary dimensions of “developing new self-esteem” and “using their diagnosis for own needs to obtain further resources”, which are both conditions described as complementing each other but also can build on one another.

*“But, an important part for me is that I simply no longer have the claim to have to do justice to everyone. Now, I am allowed to, maybe it is also a, I don’t know, hiding behind the disease, but now, I can also say, you do it.*” *(ID 03, PlwD,* “*resilience (coping)”).*

### 3.4 Contradictory conditions and strategies of the participants in content interrelations

The main dimension “communication of diagnosis” (cluster “health”, *n* = 7) was not addressed by the public stakeholders; however, implicit statements were made regarding this topic. The dimension “communication of diagnosis” reveals surprisingly contradictory strategies between perspectives, i.e., within a participant group and/or within a person. Interestingly, “masking the disease” but also, in contrast, “openness about the disease” were mentioned as strategies used to increase the feeling of safety. One PlwD mentioned positive experiences with openness and the lack of knowledge of friends and public stakeholders (e.g., employees) (ID 04). The participants reported that they constantly experienced inner conflicts.

*“And - and then, I have actually had the experience, yes, if you - and then, I have too; it’s actually not that bad. And anyway, the next time I felt it was worse again. As soon as you basically stand by that - or I stand by that - it becomes easier. And still, I have it with, and still, next time it’s a start again. - Haven’t I internalized it yet, or whatever it is*” *(ID 04, PlwD,* “*communication of diagnosis*” *and* “*social interaction”)*

This person referred to the process of the “internalization” of addressing the diagnosis of dementia, which may be an indication of the person’s coping process; however, this process was not further defined (ID 04).

Another person with dementia stated that the knowledge of friends regarding the diagnosis and the course of the disease provides a feeling of safety and is highly relevant (ID 03). In the interview, it becomes clear that the person was more active in coping with the diagnosis with the help of a social support organization as follows:

“I have - also a lot of things with them [a non-statutory welfare association], I think, I have worked through quite well for myself” (ID 03, PlwD).

In addition, it must be considered that this person is approximately 20 years younger than the person described above. This finding indicates that the characteristics and topics of several clusters can be interrelated. This finding also shows that the cluster “health” is related to the dimensions of the cluster “social togetherness” in terms of “social interaction”.

The relatives described that openness simplifies everyday situations (ID 24). However, the relatives also do not want to be confronted with stigmatization in society (ID 02). The relatives are aware of the potentially contradictory opinion of their relatives with dementia (ID 24).

*“I feel safer in any situation because I walk over it openly. I don’t hide it. It’s different now, it’s my mother, no, it’s not me. As a person with dementia you might see it differently. Because you don’t want to attract attention, just the way you are then” (ID 24, relative,* “*communication of diagnosis”)*

Further contrary strategies can be identified in terms of staying at home and self-efficacy. A trade-off exists between the decision to leave PlwD in their own environment and the decision to change their housing situation. Such decisions include the awareness that PlwD do not feel safe when participating in public life (ID 03). However, they also need to know that retreating into domesticity is helpful in the short term (ID 03, 04) but leads to problems in the long term (ID 04).

*“ (...   ) I prefer to shut myself away and I would, sometimes I would prefer to lie down in bed all day. (* ...  *) The more I shut myself away, I know that, the worse - the less I get along with my surrounding” (ID 04, PlwD, implicit data)*

In the context of self-efficacy, a trade-off exists among control by others (F2), the uncertainty PlwD feel in everyday life and the positive feeling PlwD experience when making achievements (ID 03, 02, 40). Strategies that show a compromise were also mentioned (ID 03, 21).

*“I always try to do this in such a way that she herself is in charge of it, so that she doesn’t - thinks she can’t. And, and she, she goes, she goes to breakfast and then, then she makes herself, there is buffet there, everything. Sometimes it’s, sometimes it looks a bit weird. You have to be above it when someone looks at you and doesn’t know what it’s all about, but - everything, everything’s great.” (ID 02, relative,* “*support”)*

### 3.5 Conditions and strategies from the perspective of PlwD and content variety

We observed that not all main dimensions could be addressed by the subdimensions in all participant groups. Specifically, differences were observed between the PlwD and the other participants. No subdimensions in the main dimension “social interaction” could be specified for the PlwD. Additionally, the “process of diagnosis” (*n* = 6) was only addressed in the focus group, and the cluster “society” could not be coded among the PlwD. However, some dimensions were related specifically to the PlwD. In the cluster “personal condition”, the main dimension “home as a space for personal preferences” (*n* = 5) was only addressed by the PlwD and in the focus group. The PlwD stressed the importance of the “opportunity to live according to own preferences” and “time flexibility to suit own preferences”. Considering the other participant groups, “home as a space for safety” (*n* = 18) was not addressed by the public stakeholders, and “home as a space for personal preferences” was not addressed by the relatives.

A prominent main category in the cluster “health” was the main dimension “perceived changes” (*n* = 30). Here, more strategies than conditions could be described, and most strategies were provided by the PlwD. Interestingly, the conditions showed a poor variety, exclusively emphasized the inner perspective of PlwD in terms of psychological, philosophical and theological reflections and concentrated on “personal attitudes”, “increased trust in God”, and “perception of fully functional human sense”.

*“I’m starting to calm down. It makes me feel safe. This constant nervousness about doing justice to everyone, I no longer have that demand.” (ID 03, PlwD,* “*perceived changes”)*

Although no outer conditions were mentioned, a large variety of strategies were used to address perceived chances in the context of dementia from several perspectives. Some strategies could be labeled proactive in terms of “planning situations”, “adapting structures by considering the needs of PlwD” and/ or “hiding impairments”, and some strategies could be labeled passive in terms of “avoidance” and/ or “staying at home”. The PlwD mentioned both proactive and passive strategies.

The main dimension “process of diagnosis” (cluster “health”) showed less variety in the content and was almost exclusively addressed by the participants in all focus groups but surprisingly not by the PlwD, relatives, and public stakeholders in the individual (dyadic) interviews; this dimension contains the condition “contact with competent professionals” and an interrelated strategy, e.g., “dementia-specific education for professionals”. Here, the aspect of learning was also observed. More variety was observed at the micro, meso and macro levels as follows: one strategy addressed “self-diagnosis”, one strategy mentioned professional contact and one strategy referred to a higher level of “politics” in general.

### 3.6 Factors influencing emotional safety in the context of dementia

Factors (*n* = 137 subdimensions) were identified as possible conditions influencing emotional safety in a complex way. All groups of participants described several factors, such as dementia-related, psychosocial, biographical, physical, and economic factors. In general, the influencing factors related to dementia included “stage/symptoms of dementia” (ID 01, 03, 24, 26, F1, F2, F3, FB), “symptom recognition” (ID 02), “the time since diagnosis” (F1), “confrontation with impairments/symptoms” (ID 01, 03), “knowledge (regarding dementia symptoms/diagnosis)” (ID 04, 24, F2), and “challenging behavior of PlwD” (F2).

*“So, in the first phase, (...) I didn’t even realize that she couldn’t write and couldn’t read anymore (...) Then, I practiced with her. All the stupid stuff. I thought I was doing something good for her. I wasn’t doing good for her. Well, I might have made her insecure.” (ID 02, relative,* “*confrontation with symptoms”).*

The participants mentioned psychosocial factors, e.g., “personal attitudes” (ID 02, 04, 21, 22, 25, 40, F1, F2, FB), “fear of negative reactions from others” (ID 03, 04, 40), “negative picture of dementia” (ID 02), “stigmatization” (ID F2), “perceived anonymity” (ID F2), and “inter-role conflicts” (ID 24, 26). In the context of the dimension “perceived changes”, the “interplay of psychosocial and dementia-related changes” could be identified. Aspects of support, e.g., “missing support” (ID 24, F2) or “rejection of support” (ID 21), were also important. Regarding biological factors, “characteristics of PlwD and others” (ID 02, 21, 22, 25, 26, 40, F1, F2, F3, FB), “common history” (ID 02), “parental upbringing” (ID 40), and “loss of former life” (ID 21) were influencing factors. The physical factors “environmental changes” (ID F2, F3), “physical distance to relatives” (ID 26, 39), and “missing technical solutions for PlwD” (ID F2) were highly important. Economic aspects were mentioned in general but not in a dementia-specific context as follows:

“Exactly, that is, of course, that you are healthy, that you have enough money to live, yes, and a - roof over your head, that also gives safety.” (ID 25, relative)

### 3.7 Constructs related to emotional safety

The following common constructs related to emotional safety at the psychological, psychosocial, and physical levels were reported by all or most participant groups: participation (ID 01, 02, 03, 21, 22, 24, 26, 37, 38, 39, 40, F2, F3), fear (ID 02, 03, 04, 21, 22, 24, 26, 37, 38, 39, F2, F3, FB), feeling of loneliness/togetherness (ID 21, 22, 24, 26, 37, 38, 39, 40, F2, FB), well-being (ID 03, 24, 25, 40, 41, 42, F1, F2, F3), stress (ID 21, 25, 26), and physical safety (ID 02, 21, 26, 37, 38, F2). In addition, the PlwD addressed the following constructs: harmony (ID 01), being calmer (ID 03), happiness (ID 03), self-efficacy and self-control (ID 01, 03, 40), feeling threatened (ID 37, 38), mobility (ID 37, 38), boredom (ID 37, 38), nervousness (ID 03), and being able to learn and give up tasks (ID 01, 03). The relatives reported the following additional constructs: feeling of being in good hands (ID 26), openness (ID 02, 24), being accepted (ID 02, 24, 25), being reserved (ID 02), being avoidant (ID 02), sorrow (ID 02, 24, 26), depression (ID 25), aggression (ID 25), panic (ID 26), desperation (ID 26), comfort (ID 02), and helplessness (ID 26). The public stakeholders reported the following additional constructs: feeling complete (ID 22), sleeping well (ID 21), experiencing a loss of control (ID 21), overload (ID 21), being proud (ID 22), and living in conflict (ID 21). Based on the data of the focus groups, the following additional constructs were identified: underload and feeling ashamed (ID F1).

## 4 DISCUSSION

Our study aimed to obtain a multiperspective and comprehensive view of emotional safety in the context of dementia in the living environment. The core results showed that emotional safety is a complex phenomenon. All dimensions are characterized by not only several perspectives but also a complex interrelation of different conditions, strategies, and changing influencing factors at the micro, meso, and macro levels. The overarching dimension “living and learning in relations” was developed.

These results extend existing approaches and previous findings [1, 11, 26] by broadening perspectives, considering and comparing different perspectives, and analyzing the links among the dimensions. A similar multiperspective approach is described in another study investigating dementia considering an understanding of the unique relationship dynamics within each triad (PLWD, carers and health care professionals) [26]. By considering the perspective of public stakeholders, we expanded this triadic approach.

We observed that emotional safety was highly important to all participants in different settings in daily living in the context of dementia. We were able to describe the conditions and strategies needed to ensure a feeling of safety. Other authors have also investigated emotional safety but from the single perspective of PlwD [8, 27], relatives of PlwD [11], or health care providers [28].

Within all five clusters, we observed several common, complementary, contradictory, and varying con-ditions and strategies from a multiperspective point of view. Similar to our central dimension “living and learning in relations”, in a meta-synthesis of the social relations of PlwD, Eriksen and colleagues (2016) identified a dimension described as “living a meaningful life during relational changes”. These authors described that changes in life lead to changes in relations and vice versa [29]. Furthermore, we observed that PlwD must address an “interplay of psychosocial and dementia-related changes”. Relationships and their relation to the topic of dementia are relevant. Given the “model of inner security”, relationships can be experienced as enrichment and support through safety, allowing PlwD to personally develop [8].

The importance of a public discussion regarding the topic of dementia is also emphasized by Klie in German (2015), who considers the need to strengthen the perspective of PlwD and create a living and public environment that allows an open view of the topic of dementia; however, this author also stresses that the environment allows a feeling of safety with caring social contacts [30]. The component of the “learning” dimension aims to enhance emotional safety from each perspective by considering individual and collective learning, situations of daily living, relationships and their changes over time, and the ability to adapt one’s behavior without losing one’s individuality. Based on our results, this component requires a continuous learning process from all perspectives. Other studies have shown that especially among PlwD at an early stage, it is important to provide opportunities to learn from each other (e.g., support groups) [8, 31]. However, learning is also relevant to the family of PlwD and others. Learning needs should be considered [31].

We also observed that the individual main dimensions in relation to our central dimension were related to each other, and we extend related findings. For example, Häikiö et al. (2019) showed only three discrete dimensions of preventing the emotional harm of PlwD from relatives’ perspective (“maintaining respect and dignity”, “preventing loneliness and other negative feelings”, and “creating good moments and positive feelings through activity”) [11]. Our results showed that safety needs may be expressed and perceived differently across target groups.

In particular, PlwD express the need for safety through the need for a surrounding structure. For example, Wang et al. (2012) and Wang et al. (2015) describe how PlwD express the need for safety through hoarding. This behavior can also be perceived differently [32, 33].

We further observed contradictory strategies. For example, both masking the disease and openly communicating about the disease are strategies used by PlwD to maintain a feeling of safety in social contact. These contradictory strategies were also reported by Werezak and Stewart (2002). These authors showed that the strategies are related to the characteristics of the phases addressing the disease. During the anticipation phase, PlwD can feel “anxious” regarding their social environment and the responses of others to their dementia symptoms [31]. During the symptom appearance stage, confronting situations that, in some cases, PlwD and their relatives want to avoid can occur [31]. Thus, the selection of the strategy can be influenced by several factors, e.g., cognitive processing of the disease and anticipated and previous experiences [31]. We observed these facts in our identified influencing factors, e.g., knowledge regarding dementia/diagnosis, the fear of negative reactions from others, and confrontation with symptoms (ID 03, 04, 40). Alsawy and colleagues (2020) also reported that PlwD are aware of their social interactions and recognize the negative attitudes of others [34]. The authors describe the importance of others empathizing with and understanding individuals’ situation, which can create “communication that was felt to be meaningful” for PlwD [34]. Additional strategies can be related to a decision, e.g., moving to another community [31]. Here, we also observe interrelations among our identified clusters “social togetherness”, “health”, “personal condition”, “society”, and “physical environment”.

In general, emotional safety can be influenced by dementia-related, psychosocial, biographical, physical, and economic factors. Psychosocial factors (e.g., inter-role conflicts) are an additional subdimension of influencing factors described in our previous systematic review [1]. This finding underpins our findings of a complex view of the phenomenon.

### 4.1 Constructs related to emotional safety

Finally, we obtained complex insight into common constructs related to emotional safety at the psycho-logical, psychosocial, and physical levels, e.g., participation, fear, feeling of loneliness/togetherness, well-being, stress, and physical safety. In a concep-tual analysis of the feeling of safety among pat-ients during non-dementia-specific inpatient hospitalization, Mollon (2014) described that the main consequences resulting from being in a state of emo-tional safety are “control”, “hope”, and feeling “re-laxed or calm” [35]. In our results, we also observe the experience of loss of control, self-control, and a feeling of being calmer.

### 4.2 Strengths and limitations

Although we were able to obtain a multiperspective and comprehensive picture of emotional safety in the context of dementia, there were some limitations regarding our sample design, data collection, and coding procedures. The PlwD and other participants were recruited from community services to obtain a more representative sample and consider different experiences with relevant actors in the local context. The sample of the individual interviews was balanced; however, a lower number of PlwD participated in the focus groups and feedback group due concerns related to having dementia, although beforehand, several efforts were exerted to support the PlwD. However, one PlwD exclusively participated in the focus group accompanied by his/or her relative. The recruitment was based on a criterion-based convenience and snowball-based sample. Additionally, in some cases, it was difficult to differentiate between the perspectives of the participants because the public stakeholders could also be relatives.

The aim of the study, i.e., to explore emotional safety, was explained to the interviewees several times, and we addressed the topic explicitly, which could have influenced the interviewees’ answers in this direction. However, a strength of our study is that we analyzed the differentiated data regarding what was mentioned explicitly in relation to emotional safety and what was mentioned implicitly in the interviews. We observed that the findings agreed in principle, and the findings were verified in a feedback group. In addition, we compared our results using participatory data and found that the results were similar but on different levels of abstraction.

The interviews and feedback group were performed by two interviewers. To ensure the interview quality, all interviews were performed based on a standardized procedure, and the first interviews were pretested, supervised, and discussed.

The initial coding was performed by one coder. Therefore, the reliability of the results could be res-tricted. However, the coding procedure was supervised, and all codes were repeatedly checked against the transcription sections by a second coder, dis-cussed and adapted. The data synthesis was performed by two researchers. Finally, a third researcher checked the coding trees and provided recommendations for adaption. We observed that not all main dimensions contained subdimensions in all participant groups. We discussed this fact and observed that similar subdimensions were coded in other dimensions; thus, the subdimensions that best fit the meaning in the context of the interview section and interview overall were considered.

Although generalizability of the findings is limited due to the qualitative design, we were able to reach data saturation, based on the results from different data sources including data from the participatory approach.

### 4.3 Conclusion

Our study highlights the relevance of the topic of emotional safety in the context of dementia considering care provision, services, and research from a multiperspective point of view. We found that emotional safety is a crucial need of PlwD, relatives of PlwD, and public stakeholders. The results allow more differentiated empirical picture of people’s needs for emotional safety in the context of dementia. We used a multiperspective point of view to show that there were several common, complementary, contradictory, and varying conditions and strategies that were related to individual situations, needs, and influencing factors. Considering “social togetherness”, “health”, “personal condition”, “society”, and the “physical environment” in light of “living and learning in relations” is required for care provision and services that respond to the need for emotional safety in the context of dementia and the development of interventions. In developing and choosing strategies to enhance emotional safety, complex and individual situations at the micro, meso, and macro levels in each target group should be considered in relation to each other. In consequence, care provision and service should be more integrative and be based on a consideration of emotional safety in the context of dementia. PlwD feel safer when they are seen as meaningful and esteemed persons who are learning and living in relations of their living environments, including the care processes, services, and society. The developed dimensions allow a new, structured way to find solutions for achieving emotional safety in defined settings, as well as a consideration of steadily changing social and physical settings. These solutions should include (collective) learning on all levels without the loss of individuality.

Further research should focus on the investigation of the relevance of the developed dimensions in other health care settings, e.g., stationary settings or digital settings (e.g., telehealth physician – PlwD communication) to determine how the needs of PlwD, relatives of PlwD, and public stakeholders are considered and how living and learning in relations has been or can be realized to improve emotional safety. It should be investigated how these dimensions and the way of thinking about care provision and services can best be implemented in or transferred to different settings also considering aspects of patient safety.

The proposed dimensions could be used to develop a framework for measuring emotional safety in the context of dementia. A dimension-based modular instrument could be developed to capture multiple perspectives in a structured way to determine needs, conditions, and targeted strategies.

## Supplementary Material

Supplementary Material
