# Supplementary material for: Emotional Safety in the Context of Dementia: A Multiperspective Qualitative Study
Source: J Alzheimers Dis. 2021 Jan 5;79(1):355–75. doi: 10.3233/JAD-201110 (PMC7902974; doi:10.3233/JAD-201110)
Supplement: Supplementary Material [file jad-79-jad201110-s001.pdf]

Supplementary Material

Emotional Safety in the Context of Dementia: A Multiperspective Qualitative Study

Supplementary Table 1. Interview guides (nonvalidated version translated into English for publication)

Target group	Prompts	Keywords (ask if not mentioned)	Detail-orientated elaboration and clarification probes (mandatory phrasing)
		**INTRODUCTION**	
PlwD	What does your daily routine look like after starting the day?<br>What are activities that you do not perform every day?	▪ Occasions to leave the house<br>▪ Localities<br>▪ Situations<br>▪ Activities<br>▪ People<br>▪ Items<br>▪ Moments in time	Please describe a day when you leave the house.<br>Please describe what you are doing when …
Relatives/Public stakeholders	In which situations does the topic of dementia arise?<br>In which non-daily situations does the topic of dementia arise?	▪ Localities<br>▪ Activities<br>▪ People<br>▪ Items<br>▪ Moments in time	Please describe a day when you leave the house with your relative (PlwD). What role does dementia play? *(Relatives)*<br>Please describe a day when you leave the house. What role does the topic of dementia play? *(Public stakeholders)*<br>Please describe what you are doing when …
		**MAIN PART**	
All target groups	*Ask 1 and 2 in relation to each other:*<br>1. What role does feeling safe/unsafe play in your daily routine?<br>2. What do you need to feel safe or safer?	Refer to the topics mentioned in the introduction …<br>▪ Localities<br>▪ Situations<br>▪ Activities<br>▪ People<br>▪ Items<br>▪ Moments in time	*Ask 1 and 2 in relation to each other:*<br>1. What/who provides you with a feeling of safety? When/where do you feel safe? Are there situations/activities in which you felt safe in the past but do not today?<br>2. What are you doing to feel safe? What can others do to make you feel safe? What can be done in the community to make you feel safe? When? Where? How?
		**CLOSURE**	
All target groups	Is there anything else related to the topic of feeling safe that is important to you?		
Expression of thanks *(Farewell and announcement of the feedback round)*			

Before starting the interview
□ Participant code (one code per participant during the whole project)
□ Welcome and provision of the aim and information regarding the interview and the topic of emotional safety in the context of dementia based on the information in the consent document.
□ Ongoing consent?
□ Indication and consent to record?
During the interview (paraphrasing, elaboration and clarification probes)
□ Can you tell more about…?
□ What do you mean by…?

Supplementary Table 2. Interview guide for the focus groups (translated version for publication)

Target group	Prompts	Keywords (ask if not mentioned)	Detail-oriented probes (mandatory phrasing)	Materials/additional information
		**INTRODUCTION**		
Each participant	I would like to know what you are interested in regarding the topic of feeling safe in relation to dementia.			*Each participant has the opportunity to speak, and everyone is free to decide if he or she would like to provide personal details.*
Each participant	Each participant can choose a prepared card with a topic related to the topic of feeling safe in relation to dementia.	▪ Localities<br>▪ Daily activities<br>▪ Leisure time (e.g., trips, holidays, sport)<br>▪ Traffic<br>▪ Aids/ technology<br>▪ Personal contacts<br>▪ Work<br>▪ Diagnosis *of* dementia<br>▪ Home<br>▪ Free text card		*A card system is used with the participants*
		**MAIN PART**		
All target groups	What role does the topic on your chosen card play in feeling safe in relation to dementia?<br>What would you recommend to feel safer?		What/who provides you with a feeling of safety?<br>When/where do you feel safe?<br>Are there situations/activities in which you felt safe in the past but do not today?	*Each participant can decide when to speak and is asked to name the topic on his or her card before speaking to inform the others.*
	**CLOSURE**			
All target groups	Is there anything else regarding the topic of feeling safe that is important to you?			
Expression of thanks *(farewell and announcement of the feedback round)*				

Before starting the interview:
□ Participant code (one code per participant during the whole project)
□ Welcome and provision of the aim and information regarding the interview and the topic of emotional safety in the context of dementia based on the information in the document of the consent.
□ Ongoing consent?
□ Indication and consent to record?
During the interview (paraphrasing, elaboration and clarification probes)
□ Can you tell more about…?
□ What do you mean by…?
